# Bone-Active Medication Utilization from 2013-2017 Amid Beneficiaries Aged 65+ with Medicare Part D by Provider Type

**DOI:** 10.1093/geroni/igab046.2321

**Published:** 2021-12-17

**Authors:** Jennifer Kirk, Sean Fleming, Denise Orwig

**Affiliations:** 1 University of Maryland, Baltimore & Baltimore County, Herndon, Virginia, United States; 2 University of Maryland Baltimore, University of Maryland Baltimore, Maryland, United States; 3 University of Maryland, Baltimore, Baltimore, Maryland, United States

## Abstract

As the United States’ population increasingly consists of older adults aged 65+, an increase is expected in the prevalence of osteoporosis and the number of osteoporotic fractures. Bone-active medications (BAM) delay osteoporosis progression and prevent fragility fractures, but historically low treatment persistence rates and drug utilization for BAM exist among at-risk older adults. This research assessed for differences in the BAM utilization rates over five-years in Medicare Part D by provider type: geriatric specialists (GERO), generalists, specialists, nurse practitioners (NP), and physicians’ assistants (PA). This longitudinal retrospective analysis included providers with at least one BAM prescription among beneficiaries aged 65+. An analysis of response profiles was used to model the mean BAM utilization rates overall and by provider group. Of the 50,249 providers included in this analysis, 88.15% were generalists, 5.76% specialists, 1.48% GERO, 2.73% NP, and 1.87% PA. From 2013-2017, the prevalence of BAM utilization was 6%. Over the five years, BAM utilization rates did not change significantly, but provider-specific rates were significantly different (F=12.53, p<.001). Provider-specific utilization rates were inconsistent with the highest utilization rates and most considerable variation observed among specialists (14.95%). PAs and NPs’ BAM utilization rates were stable at around 9.02% and 9.20%, but GERO and generalists exhibited the lowest utilization rates, 4.86% and 5.79%, respectively. While specialists had the higher-than-expected utilization rates, the overall and provider-specific BAM utilization rates were low and did not increase over time. Further research is needed to identify how provider-related factors, like geographic region and clinical training, influence underutilization.

